# Improvement and evaluation of the 50th percentile female prototype rear impact dummy, BioRID P50F

**DOI:** 10.3389/fbioe.2026.1837079

**Published:** 2026-07-29

**Authors:** Anna Carlsson, Johan Davidsson, Mats Y. Svensson

**Affiliations:** 1 Chalmers Industriteknik, Gothenburg, Sweden; 2 Vehicle Safety Division, Chalmers University of Technology, Gothenburg, Sweden

**Keywords:** crash test dummy, females, rear impact, sled testing, soft tissue neck injury, vehicle safety, WAD, whiplash

## Abstract

Whiplash injuries due to rear impact vehicle collisions remain a significant global concern, with females facing a disproportionately higher risk of permanent medical impairment compared to males. Current rear impact testing predominantly uses 50th percentile male dummies, such as the BioRID II, which are not representative of the female population. A 50th percentile male dummy roughly corresponds to a 90th–95th percentile female in stature and mass, which appear to render whiplash protection systems less effective for women. In preparation for future investigation of car models with known differences in whiplash injury risk between female and male occupants, the development of an upgraded average size female BioRID prototype, denoted P50F_V2, was initiated to further improve the first female BioRID prototype. The aim was to better represent female properties by reducing upper torso stiffness and improving spinal curvature, as well as simplifying the H-point position measurement and refining the external contours of the head, hands and feet. The dynamic response of the upgraded prototype was evaluated with regard to rear impact sled tests, comprising female volunteers close to the 50th percentile female size. Overall, the dynamic response of the upgraded prototype showed improvements, in particular, the T1 rearward angular displacement better matched the female volunteer response corridors. Although the biofidelity of the upgraded prototype leaves room for further improvement, it is deemed sufficient to provide an understanding of the differences in dynamic response between female and male occupants in rear impacts. Overall, the upgraded BioRID P50F, used as a complement to the BioRID II, is a significant step towards sex-inclusive crash testing.

## 1 Introduction

Vehicle crashes resulting in Whiplash Associated Disorders (WAD), commonly denoted whiplash injuries, continue to be a global concern. Although progress has been made in automotive safety–such as the introduction of Advanced Driver Assistance Systems aimed at reducing rear impacts–whiplash injuries remain prevalent and problematic in both short- and long-term outcomes (Kullgren et al., 2020). As highlighted in a review by Carlsson (2012), whiplash injuries continue to pose diagnostic and biomechanical challenges despite modern mitigation efforts. According to insurance claims records (Kullgren and Krafft, 2010), the risk reduction for permanent medical impairment was approximately 30% greater for males than for females.

Today, rear impact testing is performed with 50th percentile male dummies, mainly the BioRID II, which may limit the equality aspect in the assessment and development of whiplash protection systems, since the female part of the population is not well represented. In terms of stature and mass, the 50th percentile male crash test dummy roughly corresponds to the 90th–95th percentile female (Welsh and Lenard, 2001), resulting in females not being adequately represented by the BioRID II. Previous studies show that the BioRID II matches 50th percentile male volunteer responses (Davidsson et al., 1999; 2000). However, more recent volunteer studies show that the response of 50th percentile females is clearly different to 50th percentile males (Linder et al., 2008; Carlsson et al., 2011; Carlsson et al., 2012; Carlsson, 2012; Sato et al., 2014). Similar differences were found in a comparison between the BioRID II and a prototype rear impact crash test dummy, representing a 50th percentile female (Schmitt et al., 2012). The BioRID II is thus not adequately representative of 50th percentile females. Since the male BioRID II has been validated with regard to tests with male volunteers, current seats are assessed without consideration of female properties, despite a higher whiplash injury risk in females. This limitation may contribute to whiplash protection systems being less effective for females than for males.

A recent study by Carlsson et al. (2021b) presented a prototype rear impact crash test dummy, representing a 50th percentile female, and evaluated its performance with regard to volunteer response data. The prototype dummy, here denoted BioRID P50F_V1, was developed from modified body segments originating from the average male size BioRID II. The mass and rough dimensions of the BioRID P50F_V1 was representative of an average sized female, based on the University of Michigan Transport Research Institute (UMTRI) study (stature 1,618 mm, mass 62.3 kg; Schneider et al., 1983). The prototype dummy was evaluated against low severity rear impact sled tests, comprising six female volunteers closely resembling a 50th percentile female, with regard to stature and mass. The head/neck response of the BioRID P50F_V1 prototype resembled the female volunteer response corridors.

However, the biofidelity of the BioRID P50F_V1 prototype had some limitations. The thoracic and lumbar spinal joint stiffness was likely too stiff compared to an average female, as the stiffness remained the same as in the average sized male BioRID II. Consequently, the rearward angular and x-displacements of the T1 were less for the BioRID P50F_V1 prototype in comparison to the female volunteers. In addition, a 28%–96% greater head-to-head restraint (HR) horizontal distance (backset) was reported for the BioRID P50F_V1 compared to the BioRID II by Schmitt et al. (2012). This is in contrast to previous volunteer tests in standard vehicle seats, showing shorter backset for 50th percentile females than 50th percentile males (Welcher and Szabo, 2001; Jonsson et al., 2007; Linder et al., 2008; Carlsson et al., 2011; Carlsson et al., 2012; Carlsson et al., 2017). The greater backset of the BioRID P50F_V1 may be a result of its thoracic spinal curvature, which was taken directly from the male BioRID II dummy. Sato et al. (2016) observed that the female thoracic spinal curvature is far less kyphotic compared to the male. This suggests that the BioRID P50F_V1 T1 vertebra position is too far forward compared to an average female.

The objective of the present study was to develop an upgraded average sized female prototype dummy, the BioRID P50F_V2, based on the earlier BioRID P50F_V1. This included decreasing upper torso stiffness, improving spinal curvature, simplifying the H-point position measurement, as well as refining the contours of the head, hands and feet. This work was done in preparation for a series of six rear impact sled tests with the 50th percentile female prototype BioRID P50F_V2 and the 50th percentile male BioRID II dummies, including production seats with known injury risk (Carlsson et al., 2026).

## 2 Materials and methods

**FIGURE 1:**
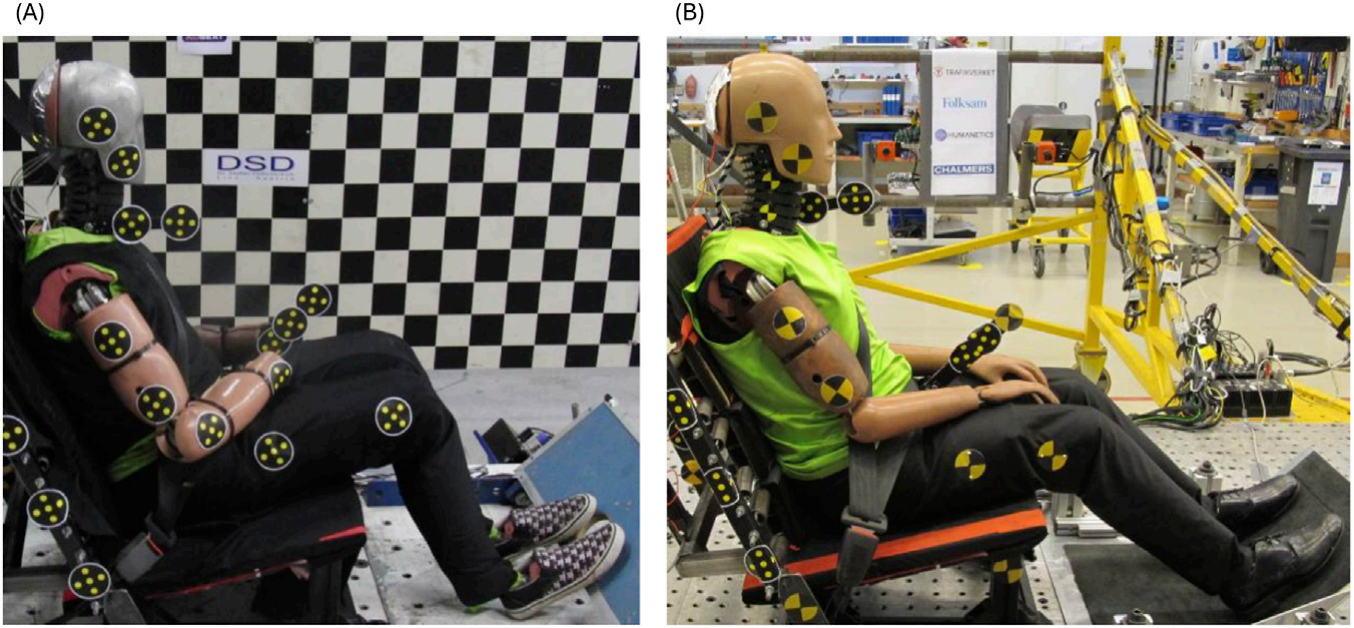
Photos of **(A)** the prototype BioRID P50F_V1 (Carlsson et al., 2021b) and **(B)** the upgraded BioRID P50F_V2, both seated in the Chalmers Laboratory Seat.

In the present study, an upgrade was made to the 50th percentile female rear impact prototype dummy, BioRID P50F_V1 (Figure 1A), previously presented in Carlsson et al. (2021b). The purpose of the upgrade to the version BioRID P50F_V2 (Figure 1B) was to 1) simplify the measurement of the H-point, 2) adjust the curvature of the spine, and 3) reduce the stiffness of the spine/torso. In addition, improvements were made to the exterior design and contact properties.

The upgraded BioRID P50F_V2, was equipped with new lower legs (including feet) as well as new lower arms (including hands). These new extremities were modified components originating from the 5^th^ percentile Hybrid III (Hybrid III 5F), providing a more natural exterior contour that improves contact conditions and provides a more human-like appearance. The same applies to the head that has now been fitted with a BioRID II head skin, giving the dummy prototype a more natural look. Furthermore, the stiffness and curvature of the spine was adjusted to closer resemble female properties and to improve the pressure distribution between the upper body and the seatback; an important prerequisite for allowing better evaluation of the backrest’s ability to evenly support the upper body and spine. The overall purpose was to obtain a prototype dummy, representative of an average female occupant for use in rear impact testing, as a complement to the male BioRID II.

The dynamic response of the upgraded prototype dummy, BioRID P50F_V2, was evaluated with regard to rear impact sled tests comprising female volunteers close to the 50th percentile female size (Carlsson et al., 2021a).

### 2.1 Upgrade of BioRID P50F_V1 to BioRID P50F_V2

#### 2.1.1 Upgrade of the head

**FIGURE 2:**
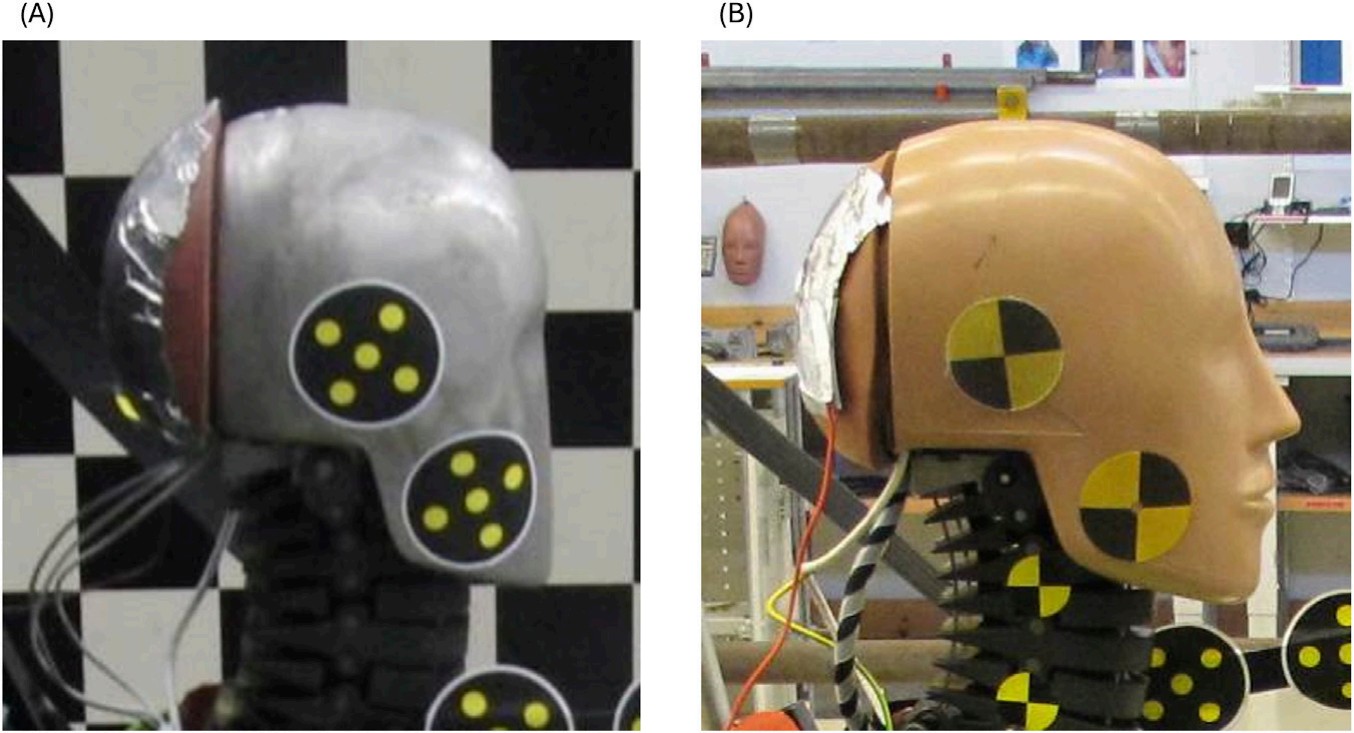
Photos of the head of **(A)** the BioRID P50F_V1 (=head from the male BioRID II with the anterior rubber skin removed; Carlsson et al., 2021b), and **(B)** the BioRID P50F_V2 (=head including the anterior rubber skin from the male BioRID II but with the ballast removed).

The head of the BioRID P50F_V1 comprised a male BioRID II head with the anterior rubber skin removed (Figure 2A). To improve this design, a BioRID II head skin was put in place, and the interior head ballast was dismantled in order to obtain a head mass representative of a 50th percentile female (Figure 2B). Once the ballast had been dismantled, the mass of the BioRID P50F_V2 head was 3.57 kg, i.e., very close to the 50th percentile female head (3.58 kg, Supplementary Table S1).

#### 2.1.2 Upgrade of the lower arms/hands

**FIGURE 3:**
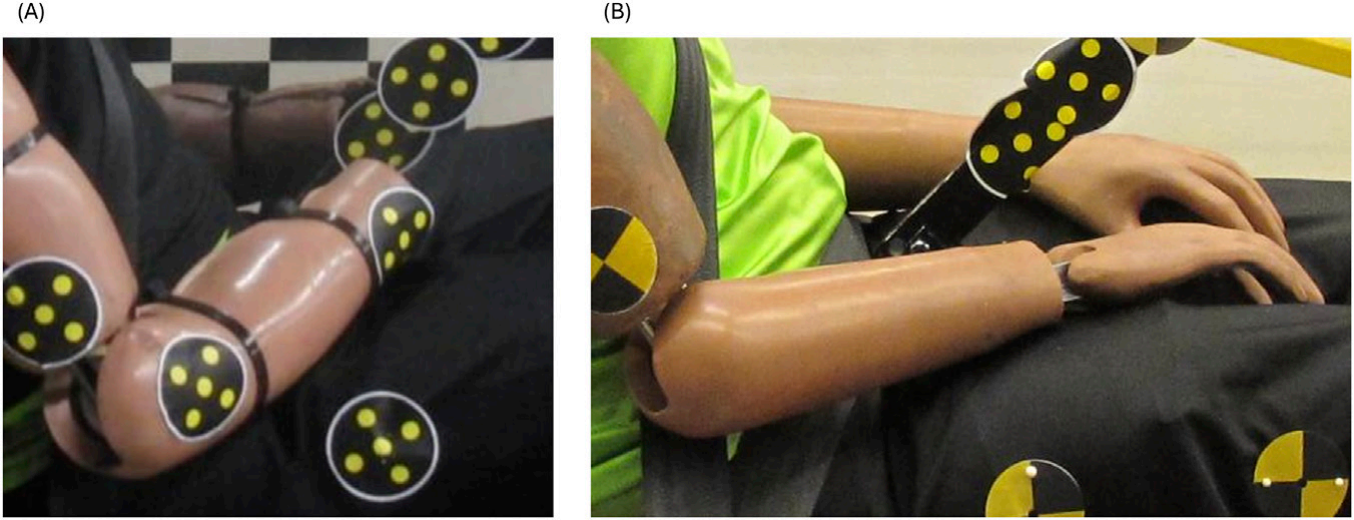
Photos of the lower arms of **(A)** the BioRID P50F_V1 (=downscaled lower arms originating from the male BioRID II; Carlsson et al., 2021b), and **(B)** the BioRID P50F_V2 (=modified lower arms originating from the Hybrid III 5F).

Target dimensions of the 50th percentile female dummy included an elbow joint-to-wrist joint distance of 234 mm and a lower arm mass of 1.16 kg including hand. The lower arms of the BioRID P50F_V1 comprised of down-scaled lower arms of the male BioRID II, shortened from 249 to 234 mm; the hands were excluded (Figure 3A). The mass of each lower arm was 1.25 kg. The lower arms of BioRID P50F_V2 comprised of modified parts from the Hybrid III 5F. Adjustments were implemented in the lower arms by increasing the length by 20 mm while keeping the mass (Supplementary Table S1). No changes were made to the hands.

In order to attach the Hybrid III 5F lower arms to the BioRID P50F_V2, the attachment of the forearm to the elbow joint was adapted to fit the dimensions of the BioRID II. The result of the modifications is shown in Figure 3B.

#### 2.1.3 Upgrade of the lower legs/feet

**FIGURE 4:**
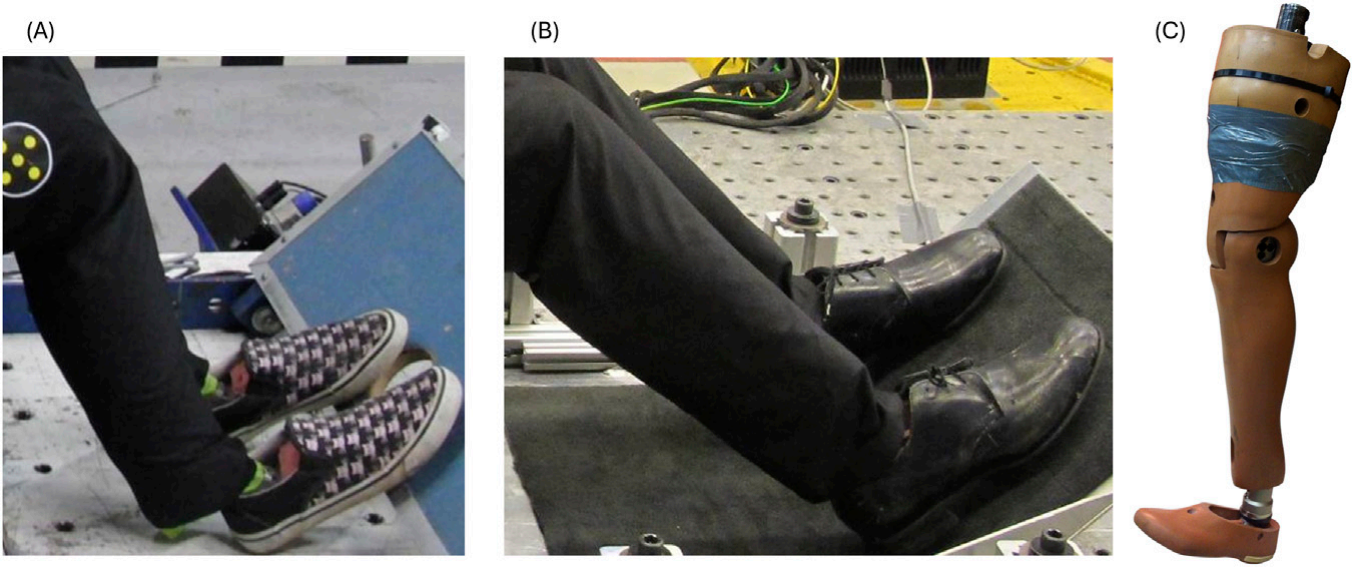
Photos of **(A)** the lower legs of the BioRID P50F_V1 (=downscaled lower legs originating from the male BioRID II, and a simple construction/ representation of the ankles/feet; Carlsson et al., 2021b), **(B)** the lower legs of the BioRID P50F_V2 (=modified lower legs originating from the Hybrid III 5F), and **(C)** the upper leg (=downscaled upper thighs originating from the male BioRID II and lower thighs originating from the Hybrid III 5F) and lower leg of the BioRID P50F_V2.

Target dimensions of the 50th percentile female dummy included a knee joint-to-floor distance of 457 mm and a lower leg mass of 3.43 kg. The lower legs of BioRID P50F_V1 comprised of down-scaled parts from the male BioRID II which–together with a simplified ankle/foot construction (Figure 4A) – resulted in a knee joint-to-floor distance of 457 mm. The lower legs of BioRID P50F_V2 comprised of modified parts from the Hybrid III 5F. The knee joint-to-floor distance was increased from 406 mm to 457 mm by extending the lower leg by 51 mm, while the mass was decreased from 4.00 kg to 3.43 kg (Supplementary Table S1). No changes were made to the feet. The result of the modifications is shown in Figure 4B.

The lower leg attachment to the thigh is the same for the Hybrid III 5F and the BioRID P50F_V2 and did not require adaptation. However, the differences in circumference between the lower part of the thigh (originating from the Hybrid III 5F) and the upper part of the thigh (originating from the BioRID II) would have created a pronounced edge. Thus, the rubber flesh in the lower part of the upper thigh was slit and held in place by a large hose clamp to reduce the circumference, and a layer of duct tape bridged the gap between the thigh units to make the transition smooth (Figure 4C). Furthermore, adding mass (0.9 kg) to the upper part of the thigh was required to equal the total mass of the leg with that of the BioRID P50F_V1. This was achieved by inserting nail shaped weights into the upper thigh, parallel to the leg.

#### 2.1.4 H-point measurement

**FIGURE 5:**
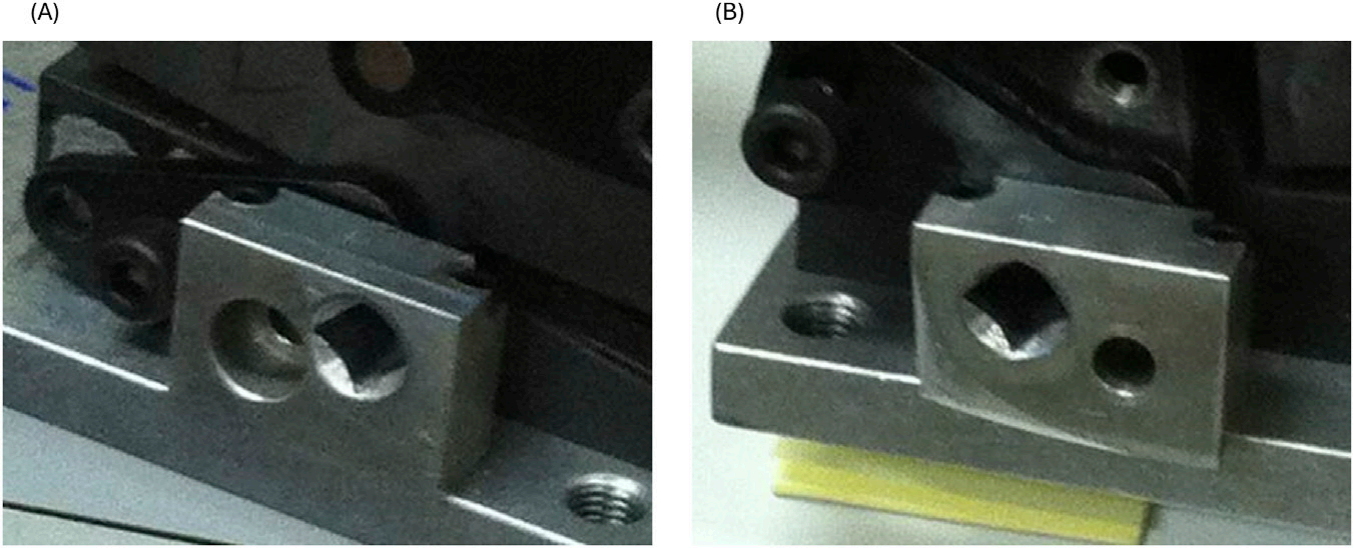
Photos of the position of the metal blocks in the BioRID P50F_V2 on **(A)** the left side and **(B)** the right side.

When constructing the BioRID P50F_V1, the lower part of the sacral vertebra (S1) was reduced by 20 mm (indicated by the dashed area in Supplementary Figure S1). Due to this reduction, two metal blocks (marked with red circles in Supplementary Figure S1), attached on each side of the spinal column, were removed. These metal blocks hold the attachment holes for the so-called H-point tool used for measuring the pelvis H-point position relative to the seat. Two new metal blocks were fixed directly to the S1 base plate (Figure 5), incorporating H-point tool attachments in the BioRID P50F_V2.

#### 2.1.5 Adjustment of the spinal curvature

Females tend to sit in a more upright position, compared to males, with their head positioned closer to the head restraint (Carlsson et al., 2017; Jonsson et al., 2007). The spinal curvature of the BioRID P50F_V1 was the same as for the male BioRID II. The only difference was that two lumbar vertebrae (L4 and L5) and 20 mm of the S1 were removed in the BioRID P50F_V1. Thus, adjusting the curvature of the spine to better represent female properties would improve the posture of the BioRID P50F_V2. Data regarding the spinal curvature for 5^th^ percentile females was retrieved from the previously performed study at UMTRI (Schneider et al., 1983; Robbins, 1983).

**FIGURE 6:**
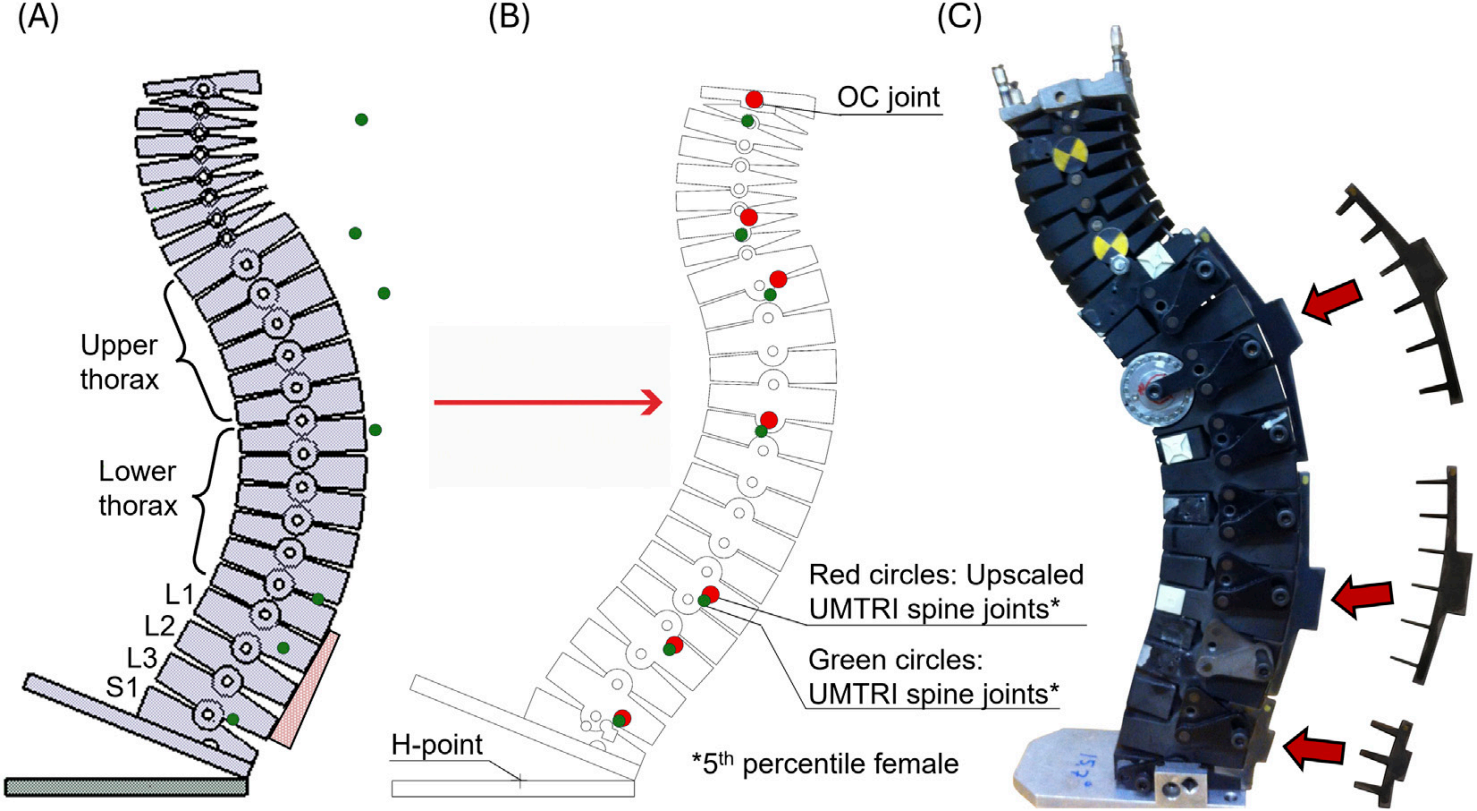
**(A)** Drawing of the spine of the BioRID P50F_V1 (Carlsson et al., 2021b), as well as articulation points (green circles) for the 5th percentile female in accordance with the UMTRI study (Schneider et al., 1983). **(B)** The spine curvature for the BioRID P50F_V2, as well as upscaled articulation points (red circles). **(C)** The spinal curvature adjustment on BioRID P50F_V2 using the tools (marked in red arrows + small figures to the right, corresponding to Supplementary Figure S2).

The spinal curvature of the BioRID P50F_V1 was modified according to Figure 6. The coordinates of eight different joint centres in the 5^th^ percentile female (Schneider et al., 1983; Robbins, 1983; Table 1) were added to a CAD model of the spine (green filled circles in Figure 6A), where the H-point was used as a common starting point. A wedge-shaped structure was inserted at the lower part of the spine to compensate for the difference in thickness of the rubber torso along the spine. The base plate was then rotated 22° rearwards to mimic the spinal angle at the normal seated posture in the car seat (based on Schneider et al., 1983).

**TABLE 1:** The spinal joint coordinates for the 5^th^ percentile female according to the UMTRI study (Schneider et al., 1983) and scaled coordinates.

Joint	5th percentile female^1)^		Scaled coordinates^2)^	
	X	Z	X	Z
Head/neck (OC)	−189	519	−195.0	535.6
C7/T1	−183	429	−188.9	442.7
T4/T5	−205	381	−211.6	393.2
T8/T9	−196	273	−202.3	281.7
T12/L1	−149	140	−153.8	144.5
L2/L3	−121	102	−124.9	105.3
L5/S1	−80	46	−82.6	47.5
H-point	0	0	0.0	0.0

1) Schneider et al. (1983). 2) Scaling factor 1.03.

To make the spinal curvature of the BioRID P50F_V2 consistent with that of the 5^th^ percentile female, the spine was divided into three sections: lumbar (L1–L3), lower thorax, and upper thorax (Figure 6A). Each section had a specific angle between the vertebrae, and adjustment of the curvature of the spine was made possible by adjusting these angles. In addition, the joint coordinates were scaled up to fit the size of the BioRID P50F_V2 (Table 1). The angular change and the upscaling of coordinates were tuned through an iterative process with the aim of, 1) aligning the occipital condyles (OC), 2) the OC angle not exceeding 5°, and 3) matching well to the female spinal curvature. These conditions were met using the following combination:

➢ Scaling factor: 1.032
➢ Lumbar angle: 5.8°
➢ Lower thorax angle: 2.1°
➢ Upper thorax angle: 4.9°

resulting in an OC angle of 4.9°. The final spinal curvature of the BioRID P50F_V2 is shown in Figure 6B. The upscaling to the 50th percentile female resulted in adjusted coordinates of the joint centres (red filled circles in Figure 6B). Based on this curvature, a tool was manufactured to adjust the angles between adjacent vertebrae of the BioRID P50F_V2 (Figure 6C; Supplementary Figure S2).

#### 2.1.6 Adjustment of spinal stiffness

Compared with 50th percentile female volunteer tests, the BioRID P50F_V1 exhibited a smaller rearward angular displacement of T1 (Carlsson et al., 2021b). Thus, to achieve closer resemblance with the female volunteer tests, the spinal stiffness had to be reduced. For that purpose, the following changes were made.

**FIGURE 7:**
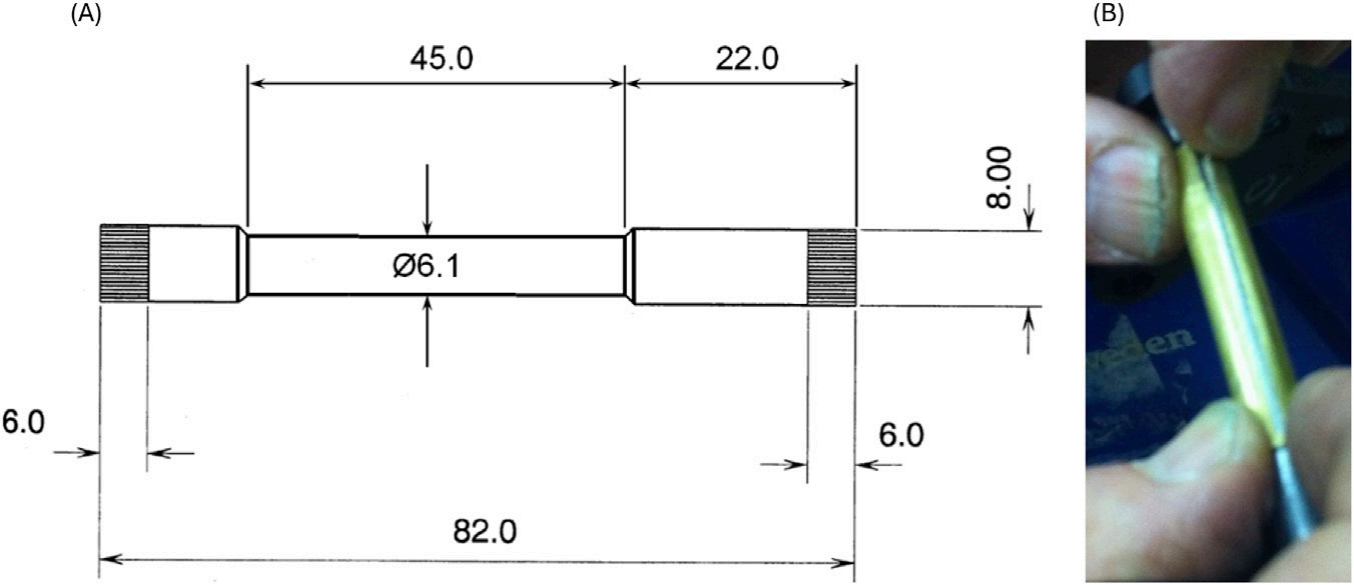
**(A)** Drawing of the modified torsion bar, and **(B)** torsion rod with associated brass sleeve.

➢ The polyurethane bumpers were removed between the nine top thoracic vertebrae.
➢ Deep cuts were introduced in the rubber torso to further reduce the torso sagittal bending stiffness (Supplementary Figure S3).
➢ The stiffness of the ten uppermost torsion rods was decreased by reducing the diameter from 8 mm to 6.1 mm (Figure 7A). To compensate for the reduced diameter, brass sleeves were placed around the torsion rods before reassembling the spine (Figure 7B).

### 2.2 Evaluation test in the Chalmers Laboratory Seat

The upgraded BioRID P50F_V2 was evaluated by performing one test in the Chalmers laboratory seat, in equivalent test conditions (Δv = 7 km/s; a_mean_ = 2 g) as in previous tests with 1) six 50th percentile female volunteers (Carlsson et al., 2021a) and 2) the BioRID P50F_V1 (Carlsson et al., 2021b) Supplementary Figure S4). The test was performed at Autoliv’s Test Track 2 (Inverse Crash System, Mannesmann Rexroth AG, Germany).

The construction of the Chalmers laboratory seat was the same as in the previous test series (Supplementary Figure S4; Carlsson et al., 2021a). The seatback was designed to resemble the shape and deflection properties of a Volvo 850 car seat, comprising four stiff panels covered in 20 mm medium quality Tempur foam, topped with a plush fabric from a Volvo car seat. The panels were independently mounted to a rigid seatback frame by coil springs to facilitate reproduction in a computational model such as that of Genzel et al. (2022). The seatback was adjusted to 24°. The head restraint comprised a plywood panel (dimensions: 350 × 230 × 20 mm) supported by a stiff steel frame. The head restraint was height adjustable, and the surface had an angle of 12.4° from the vertical plane. The initial head-to-head restraint horizontal distance (backset) was adjusted to 15 cm by adding 130 mm padding to the head restraint. The seatbase comprised a stiff aluminium frame covered by a plywood top surface, angled 16.9° from the horizontal plane. A plate was mounted on the target sled to resemble the passenger floor pan surface of a car. A three-point seatbelt was included in the test setup.

The BioRID P50F_V2 prototype was equipped with accelerometers in the head on the 1^st^ thoracic vertebra (T1). The coordinate systems were defined according to SAE J211 (orthogonal right-handed). The centres of the accelerometers’ coordinate systems were fixed on the respective accelerometer positions. The head and T1 accelerometers were mounted with initial axes coinciding with the SAE J211 standards. Additionally, the Neck Injury Criterion (NIC) values (Boström et al., 2000) were calculated. The torso leaned against the seatback, and the T1, as well as the head, were aligned with the horizontal plane. The lower arms were positioned on the upper legs.

Film targets were secured on the BioRID P50F_V2 and on the seat prior to the tests (Supplementary Figure S4). Linear and angular displacements of the head and T1 were derived from video analysis using the Tema 3.8 software. The displacement data were set to zero at the time of impact (T = 0) and were expressed in a sled fixed coordinate system.

## 3 Results

**FIGURE 8:**
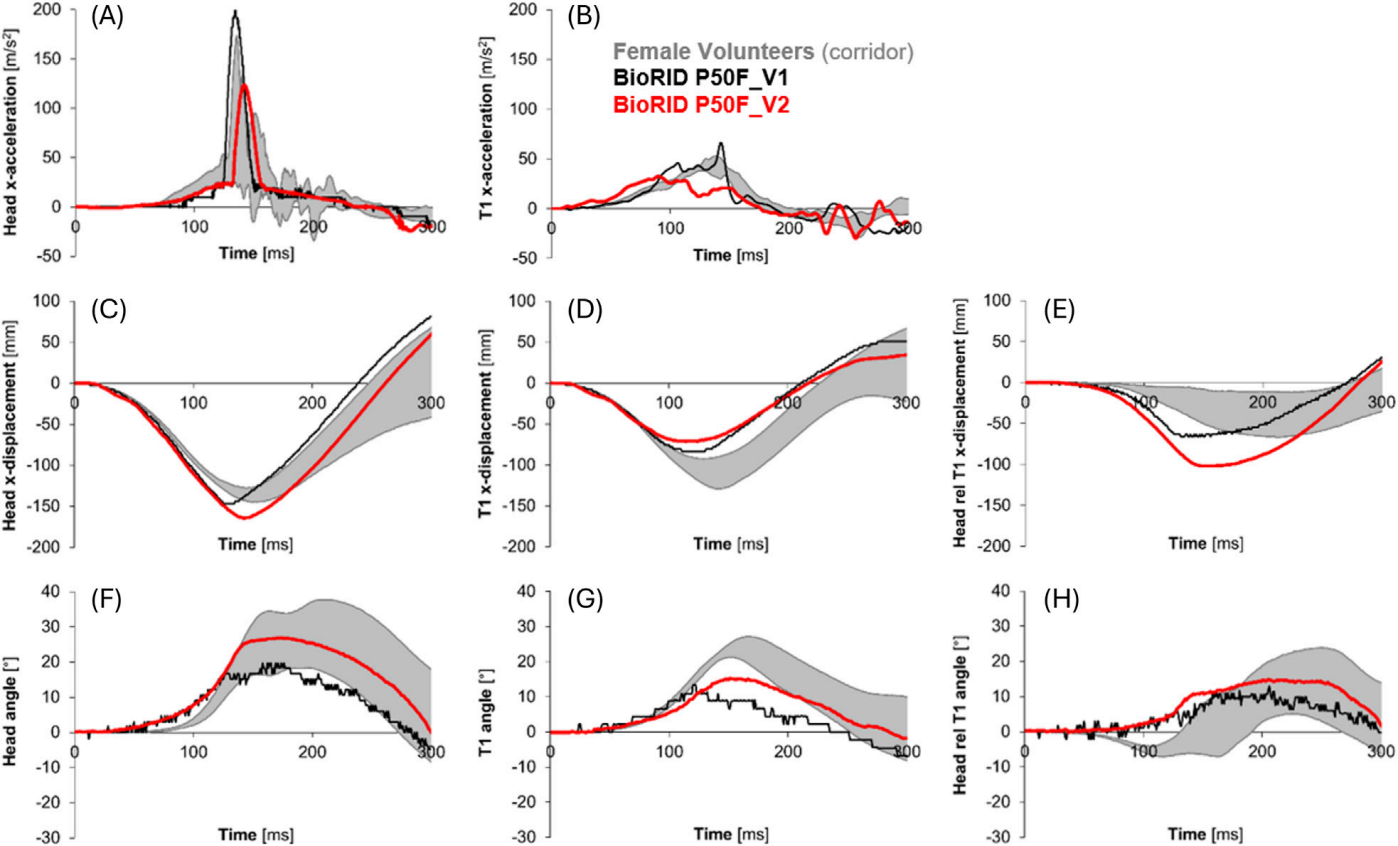
Acceleration of the **(A)** head and **(B)** T1 in the local x-direction; x-displacements relative to the sled of the **(C)** head, **(D)** T1, and **(E)** head relative to T1; angular displacements of the **(F)** head, **(G)** T1, and **(H)** head relative to T1 for the female volunteers (grey corridors), BioRID P50F_V1 prototype (solid black line) and BioRID P50F_V2 prototype (solid red line). The response corridors were calculated ±1SD from the average response and all signals were shifted to zero at time of impact initiation.

The overall responses of the upgraded prototype BioRID P50F_V2, and the previous version of the BioRID P50F_V1 (Carlsson et al., 2021b), are presented in Figure 8 in comparison to the 50th percentile female volunteers (Carlsson et al., 2021a). The volunteers are represented by grey corridors, and the prototype female dummies by black (BioRID P50F_V1) and red (BioRID P50F_V2) solid lines.

The peak head x-acceleration of the BioRID P50F_V2 was reduced in comparison to the BioRID P50F_V1 (Table 2) and the curve was mainly within the volunteer corridor (Figure 8A). The T1 x-acceleration of the BioRID P50F_V2 showed an earlier initial increase in magnitude compared with both the BioRID P50F_V1 and the volunteers. For example, the T1 x-acceleration reached 20 m/s^2^ at 61 m for the BioRID P50F_V2, compared with 84 m for both the BioRID P50F_V1 and the volunteers (on average). After ∼110 m, the T1 x-acceleration of the BioRID P50F_V2 decreased, resulting in a lower peak value (32 m/s^2^) than those observed in the BioRID P50F_V1 (67 m/s^2^) and the volunteers (average 47 m/s^2^) (Figure 8B; Table 2). Since the thoracic spinal shape was straighter in the BioRID P50F_V2 it is expected that the upper torso contacted the top seatback panel earlier. This made the total seatback response stiffer and created a greater T1 x-acceleration in the initial phase and an earlier peak. This is also reflected in the Neck Injury Criterion (NIC) value (Table 2), which decreased from 8.5 m^2^/s^2^ in the test with the BioRID P50F_V1 to 5.8 m^2^/s^2^ with the BioRID P50F_V2. This represents an improvement, as the latter value approaches the average NIC observed among the female volunteers (5.0 m^2^/s^2^).

**TABLE 2:** Summary of selected peak parameter values obtained in the tests with female volunteers, BioRID P50F_V1 and BioRID P50F_V2 in the laboratory seat.

Variable	Volunteers				BioRID P50F_V1		BioRID P50F_V2	
	Peak		@		Peak	@	Peak	@
	Average (SD)	Range	Average (SD)	Range				
X-acceleration	[m/s^2^]	[m/s^2^]	[ms]	[ms]	[m/s^2^]	[ms]	[m/s^2^]	[ms]
- Head	106 (40)	66 → 173	147 (8)	135 → 158	199	134	124	142
- T1	47 (6)	38 → 54	135 (13)	113 → 153	67	142	32	90
- Head relative to T1^2)^	−15 (2)	−19 → −13	80 (6)	72 → 89	−37	106	37	86
- Head relative to T1^3)^	71 (33)	33 → 120	147 (8)	135 → 158	158	134	38	104
X-displacement^1)^	[mm]	[mm]	[ms]	[ms]	[mm]	[ms]	[mm]	[ms]
- Head	−138 (9)	−147 → −125	149 (7)	141 → 156	−147	130	−164	143
- T1	−103 (9)	−145 → −94	136 (7)	129 → 145	−86	123	−71	124
- Head relative to T1	−52 (12)	−65 → 13	187 (36)	147 → 233	−63	132	−102	160
Ang. Displacement	[°]	[°]	[ms]	[ms]	[°]	[ms]	[°]	[ms]
- Head	28 (9)	16 → 38	202 (13)	185 → 216	20	167	27	174
- T1	24 (3)	21 → 30	159 (8)	151 → 174	13	132	15	151
- Head relative to T1^2)^	−7 (2)	−9 → −4	126 (27)	99 → 164	-	-	-	-
- Head relative to T1^3)^	15 (9)	5 → 26	235 (15)	212 → 258	12	200	15	205
NIC	[m^2^/s^2^]	[m^2^/s^2^]	[ms]	[ms]	[m^2^/s^2^]	[m]	[m^2^/s^2^]	[ms]
	5.0 (2.1)	3.0 → 7.8	123 (23)	79 → 141	8.5	106	5.8	89
Head restraint	[mm]	[mm]	[ms]	[ms]	[mm]	[m]		
- Distance^4)^	144 (6)	135 → 153	-	-	150	-	150	-
- Contact	-	-	129 (8)	118 → 139	-	120	-	131

1) Relative to the sled

2) First peak

3) Second peak

4) At T = 0 m (based on film analysis)

The rise of the head x-displacements (relative to the sled) was similar for the two prototype dummies and the peaks appeared somewhat earlier (130 m in BioRID P50F_V1 and 143 m in BioRID P50F_V2) in comparison to that of the average female volunteer (149 m). Furthermore, the BioRID P50F_V2 had a 17 mm greater negative peak head displacement in comparison to the lower limit of the volunteer corridor as well as the BioRID P50F_V1 (Figure 8C; Table 2). The T1 x-displacements were similar for the two prototype dummies, approximately 2–3 cm smaller than that of the average volunteer (Figure 8D; Table 2). As a consequence, the negative peak head relative to T1 x-displacement of the BioRID P50F_V2 was greater and earlier compared to the volunteer corridor (Figure 8E).

The peak rearward head angular displacement of the BioRID P50F_V2 increased by 7° compared to the BioRID P50F_V1 and the curve was mainly within the volunteer corridor from ∼110 m (Figure 8F; Table 2). The T1 angular displacement followed the rise of the corridor until ∼90 m and peaked at 151 m, close to the average peak time of 159 m observed in the volunteers, representing an improvement compared with the BioRID P50F_V1 (132 m) (Figure 8G; Table 2). The peak magnitude increased by approximately 2° compared with the BioRID P50F_V1, however, a difference of 6° remained relative to the volunteer corridor. The volunteers exhibited a small flexion of the head relative to the T1 angular displacement during the first ∼160 m, since the rearward angular displacement of T1 began earlier than that of the head. This small flexion was not found in any of the BioRID P50F prototypes due to the early onset of the head angular displacement (Figure 8H). As the volunteers’ heads began to rotate rearward, the flexion of the head relative to T1 changed into extension. The corresponding extension angle for the BioRID P50F_V2 prototype was within the corridor of the female volunteers.

## 4 Discussion

The overall purpose of the present study was to develop an improved female prototype dummy for rear impact testing to complement the male BioRID II. This work was done in preparation of a series of rear impact sled tests, to compare female and male occupant responses in an existing seat design, with documented differences in injury risk (Carlsson et al., 2026). The new prototype has been denoted BioRID P50F_V2 and was based on the earlier BioRID P50F_V1, which has shown some biofidelity limitations (Carlsson et al., 2021b). The BioRID P50F_V2 features new lower limbs and arms from the Hybrid III 5F, giving it a more human-like appearance and improved contact conditions. Redesigning the head with facial features has made it look more humanlike and improves external contact properties. The spinal curvature and stiffness were also modified to better reflect female anatomy and improve pressure distribution against the seatback, enabling more accurate evaluation of the seatback interaction.

The overall BioRID P50F_V2 anthropometry and mass distribution is representative of a 50th percentile female, and its biofidelity has been further improved, in particular regarding the T1 angular motion. Together with the BioRID II, it offers the opportunity to study the overall response differences between female and male occupants in rear impact testing, for example, in Carlsson et al., 2026. In a previous study (Carlsson et al., 2021b), it was concluded that the rearward angular displacement of the T1 was inadequate for the BioRID P50F_V1 prototype in comparison to the female volunteers. Therefore, obtaining increased T1 rearward angular displacement was prioritised in the present study.

The dynamic response of the BioRID P50F_V2 showed improvements, in particular the T1 rearward angular displacement (Figure 8G), which better resembled the female volunteer response corridors. The rearward T1 peak angular displacement showed improved timing, reaching its maximum at approximately the same time as the volunteers (151 m compared with 159 m) (Figure 8G; Table 2). The peak magnitude (15°) also increased but remained 6° below the lower bound of the volunteer corridor (21°). The limited peak magnitude may partly be explained by the initial position of T1 being closer to the seatback due to the changes in spinal curvature. This may also account for the somewhat reduced rearward T1 x-displacement in the BioRID P50F_V2 (71 mm) compared with the BioRID P50F_V1 (86 mm), both of which are lower than the lower bound of the volunteer corridor (94 mm) (Figure 8D; Table 2). Another reason for the limited rearward T1 angular displacement may be that the height of the T1 in the BioRID P50F prototype dummy is ∼20 mm lower as compared to the 50th percentile female, as all the downsizing modifications from the original BioRID II spine were made between the T1 and the pelvis, i.e., not the neck (Carlsson et al., 2021b).

The HR design and the positioning procedure of the lab seat used in the current study, as well as in the volunteer tests of Carlsson et al. (2021a), allowed for controlling the horizontal head-to-HR backset. This made the set-up insensitive to the individual spread in posterior-anterior position of the head that would normally show up in regular car seats. A limitation is that this validation set-up does not fully evaluate the representativeness of the initial horizontal dummy head position, hence, it is recommended that future evaluations include this aspect.

The present validation of the BioRID P50F_V2 uses volunteer test data of Carlsson et al. (2021a). Those volunteer test results only included one injury criterion, the NIC (Boström et al., 2000). Other neck injury criteria have been suggested in the literature, including N_km_, N_ij_, NDC and more (Schmitt et al., 2025). In future volunteer testing it would be desirable to include also such alternative injury criteria to the extent that they could be assessed with the type of instrumentation that can be fitted on human volunteers. One limitation of the current validation of the BioRID P50F_V2 is that it only uses data from one volunteer study with one laboratory seat design. It would be valuable to expand the validation with results from additional volunteer studies using other seat properties such as Sato et al. (2014). Another limitation is that this study relies on one single test. But we found this acceptable since the BioRID P50F_V2 is based on the same design principle as the BioRID II. Previous studies have shown that the BioRID II is a highly repeatable and reproducible dummy design (Eriksson and Zellmer, 2007).

A future next step, to advance the work undertaken in the current study, would be to develop a female rear impact dummy in which each component is based on actual female anatomy and biomechanical properties. For example, female properties of the seated spinal curvature could be obtained based on MRI scans of the average sized females of Sato et al. (2016). With the suggested spinal improvement, the rubber torso will need to be updated accordingly to fit the new spinal curvature and vertebral heights. This procedure would result in a higher position of T1 relative to the seatback and would consequently increase the rearward T1 linear and angular displacement, potentially leading to better resemblance with the volunteer test results.

In addition, this study used an average male size dummy head on the BioRID P50F_V2, but with a mass corresponding to that of an average female. The fore-aft head depth of the average female and average male dummy are 18.7 cm and 19.6 cm respectively (Supplementary Table S1). Since the initial backset was adjusted to 15 cm in each test, the difference in geometrical head size between the average female and male likely had very limited influence on the initial contact time. But, apart from the fore-aft head depth, the geometric differences between the male size dummy head and that of an average female will to some extent also affect the rear contact surface area as well as the lever arm effect on the neck structures. A future female rear impact dummy needs to take these properties into account.

As virtual testing is expected to play an important role in complementing sled tests with mechanical dummies (Ressi et al., 2025), developing and validating an FE model of the same dummy will be necessary. In turn, the updated FE model would allow for the assessment of a wider range of impact conditions. From a long-term perspective, this FE model can be morphed into a wider range of occupant characteristics and postures. This would enhance the understanding of sex and size specific responses, which mechanical dummies cannot cover. Simultaneously, physical sled tests remain indispensable for model evaluation. The integration of virtual and physical testing therefore offers a more comprehensive and scientifically robust framework for the development and assessment of future occupant protection systems.

To conclude, the BioRID P50F_V2 prototype showed a response broadly consistent with female volunteer data in low severity rear impacts. However, further refinement–particularly of surface geometry, mass distribution, and spinal geometry–is likely required to achieve biofidelity fully equal to BioRID II. The same approach as used in the development of the VIVA + human body model (John et al., 2022) can be adopted, where several statistical shape models were used as input. The need for 50th percentile rear impact dummies representing both female and male occupants is very apparent. The current BioRID II represents the 50th percentile male, and the lack of a female counterpart limits the possibility for development and evaluation of whiplash protection systems for female occupants. Preferably, both dummy sizes should follow the same design principles and offer comparable levels of biofidelity, validated against volunteer response data using metrics such as Correlation and Analysis (CORA) or similar. To ensure gender-inclusive safety, future systems must be developed and assessed with female-specific characteristics in mind. This study highlights the importance of developing a fully validated 50th percentile female rear impact dummy for use alongside the existing male dummy.

## Data Availability

The raw data supporting the conclusions of this article will be made available by the authors, without undue reservation.

## References

[B1] Boström O. Fredriksson R. Håland Y. Jakobsson L. Krafft M. Lövsund P. (2000). Comparison of car seats in low speed rear-end impacts using the biorid dummy and the new neck injury criterion (NIC). Accid. Anal. Prev. 32, 321–328. 10.1016/s0001-4575(99)00105-0 10688488

[B2] Carlsson A. (2012). “Addressing female whiplash injury protection—A step towards 50th percentile female rear impact occupant models,” in Department of Applied Mechanics. Gothenburg, Sweden: Chalmers University of Technology. PhD Thesis.

[B3] Carlsson A. Linder A. Davidsson J. Hell W. Schick S. Svensson M. (2011). Dynamic kinematic responses of female volunteers in rear impacts and comparison to previous male volunteer tests. Traffic Inj. Prev. 12 (4), 347–357. 10.1080/15389588.2011.585408 21823943

[B4] Carlsson A. Siegmund G. P. Linder A. Svensson M. Y. (2012). Motion of the head and neck of female and Male volunteers in rear impact car-to-car impacts. Traffic Inj. Prev. 13 (4), 378–387. 10.1080/15389588.2012.659362 22817553

[B5] Carlsson A. Pipkorn L. Kullgren A. Svensson M. (2017). Real-world adjustments of driver seat and head restraint in saab 9-3 vehicles. Traffic Inj. Prev. 18 (4), 398–405. 10.1080/15389588.2016.1217522 27617749

[B6] Carlsson A. Davidsson J. Linder A. Svensson M. (2021a). Dynamic responses of female volunteers in rear impact sled tests at two head restraint distances. Front. Bioeng. Biotechnol. 9, 684003. 10.3389/fbioe.2021.684003 34169067 PMC8217471

[B7] Carlsson A. Davidsson J. Linder A. Svensson M. Y. (2021b). Design and evaluation of the initial 50th percentile female prototype rear impact dummy, BioRID P50F – indications for the need of an additional dummy size. Front. Bioeng. Biotechnol. 9, 687058. 10.3389/fbioe.2021.687058 34336802 PMC8322785

[B8] Carlsson A. Kullgren A. Svensson M. Y. (2026). Rear impact responses of 50^th^ percentile female and male dummies in a seat with different whiplash injury risk reduction for women and men. Front. Bioeng. Biotechnol. Sec. Biomechanics. 10.3389/fbioe.2026.1837076 PMC13542634 42699075

[B9] Davidsson J. Flogård A. Lövsund P. Svensson M. Y. (1999). “BioRID P3 – design and performance compared to hybrid III and volunteers in rear impacts at ΔV = 7 km/h,” in Paper Presented at the 43^rd^ Stapp Car Crash Conference, October 25–27. San Diego, California, USA.

[B10] Davidsson J. Lövsund P. Ono K. Svensson M. Y. Inami S. (2000). A comparison of volunteer, BioRID P3 and hybrid III performance in rear impacts. J. Crash Prev. Inj. Control. 2, 203–220. 10.1080/10286580108902565

[B11] Eriksson L. Zellmer H. (2007). “Assessing the BioRID II repeatability and reproducibility by applying the objective Rating method (ORM) on rear-end sled tests,” in Proceedings of the 20^th^ International Technical Conference on the Enhanced Safety of Vehicles (ESV) (Lyon: National Highway Traffic Safety Administration NHTSA).

[B12] Genzel J. Carlsson A. Linder A. Pipkorn B. Svensson M. (2022). “An open-source finite element model of a generic car seat: development and validation for low-severity rear impact evaluations,” in the Proceedings of the IRCOBI Conference, 14–16 September 2022–.Porto, Portugal, 229–242.

[B13] John J. Klug C. Kranjec M. Svenning E. Iraeus J. (2022). Hello, world! VIVA+: a human body model lineup to evaluate sex-differences in crash protection. Front. Bioeng. Biotechnol. 19 (10), 918904. PMID: 35928956; PMCID: PMC9343945. 10.3389/fbioe.2022.918904 35928956 PMC9343945

[B14] Jonsson B. Stenlund H. Svensson M. Y. Björnstig U. (2007). Backset and cervical retraction capacity among occupants in a modern car. Traffic Inj. Prev. 8 (1), 87–93. 10.1080/15389580600911010 17366340

[B15] Kullgren A. Krafft M. (2010). “Gender analysis on whiplash seat effectiveness: results from real-world crashes,” in Proceedings of the IRCOBI Conference, September 15–16. Germany: Hanover, 17–28.

[B16] Kullgren A. Stigson H. Axelsson A. (2020). “Developments in car crash safety since the 1980s,” in Proceedings of the IRCOBI Conference 2020 (The Conference Was Not Held in Person, due to Concerns Related to the Corona Pandemic, but the Proceedings Are the Official Record of the Conference).

[B17] Linder A. Carlsson A. Svensson M. Y. Siegmund G. P. (2008). Dynamic responses of female and male volunteers in rear impacts. Traffic Inj. Prev. 9 (6), 592–599. 10.1080/15389580802384669 19058107

[B18] Ressi F. Schneider B. Kofler D. Kannan V. Hartlen D. C. Cronin D. S. (2025). “Development of a robust human body model qualification methodology: ensuring biofidelity for occupant protection assessments in virtual testing,” in Proceedings of the IRCOBI Conference. September 10–12, Vilnius, Lithuania, IRC-25-22.

[B19] Robbins D. H. (1983). Anthropometric Specifications for Small Female and Large Male Dummies. Ann Arbor, Michigan, USA: University of Michigan Transportation Research Institute.

[B20] Sato F. Nakajima T. Ono K. Svensson M. Brolin K. Kaneoka K. (2014). “Dynamic cervical vertebral motion of female and male volunteers and analysis of its interaction with head/neck/torso behavior during low-speed rear impact,” in IRCOBI Conference (Berlin, Germany), 10–12.

[B21] Sato F. Odani M. Miyazaki Y. Nakajima T. Makoshi J. A. Yamazaki K. (2016). “Investigation of whole spine alignment patterns in automotive seated posture using upright open MRI systems,” in Proceedings of the IRCOBI Conference, 113–130.

[B22] Schmitt K.-U. Weber T. Svensson M. Davidsson J. Carlsson A. Björklund M. (2012). “Seat testing to investigate the female neck injury risk – preliminary results using a new female dummy prototype,” in Proceedings of the IRCOBI Conference, 263.

[B23] Schmitt K. U. Cronin D. S. Morrison III B. Callaghan J. P. Muser M. H. (2025). Trauma Biomechanics: An Introduction to Injury Biomechanics. 10.1007/978-3-032-00357-7

[B24] Schneider L. W. Robbins D. H. Pflüg M. A. Snyder R. G. (1983). Development of Anthropometrically Based Design Specifications for an Advanced Adult Anthropomorphic Dummy Family. Ann Arbor, Michigan, USA: University of Michigan Transportation Research Institute. Final Report, UMTRI-83-53-1.

[B25] Welcher J. B. Szabo J. S. (2001). Relationships between seat properties and human subject kinematics in rear impact tests. Accid. Anal. Prev. 33 (3), 289–304. 10.1016/S0001-4575(00)00043-9 11235791

[B26] Welsh R. Lenard J. (2001). “Male and female car drivers – differences in collision and injury risks,” in Proceedings of the 45^th^ Assoc. Adv. Automot. Med. (AAAM) Conference. Texas, USA, 73–91. 12214367

