# Supplementary material for: Improvement and evaluation of the 50th percentile female prototype rear impact dummy, BioRID P50F
Source: Front Bioeng Biotechnol. 2026 Jul 29;14:1837079. doi: 10.3389/fbioe.2026.1837079 (PMC13462242; doi:10.3389/fbioe.2026.1837079)
Supplement: Supplementary file 1 [file DataSheet1.pdf]

Supplementary Material

Upgrade of the head

To improve this design, two primary options were considered: either to modify an existing dummy head (the 5th percentile female Hybrid III (Hybrid III 5F) or the male BioRID II), or to manufacture a unique 50th percentile female head. Due to the limited project budget, the latter option was excluded. The head specifications are presented and compared to those of the 5th percentile Hybrid III dummy in **Supplementary Table 1**.

The head dimensions of the Hybrid III 5F are slightly smaller compared to the 50th percentile female (2% lesser circumference, 3% lesser breadth and 2% lesser depth), while the BioRID 50M's head is slightly larger (4% greater circumference, 6% greater breadth, 5% greater depth) (**Supplementary Table 1**). During discussions with Humanetics, manufacturer of crash test dummies, it became clear that switching to a Hybrid III 5F's head was not an achievable alternative, mainly due to the head of the Hybrid III 5F's would be in conflict with the upper neck region of the BioRID P50F_V2 (originating from a BioRID 50M). Instead, it was suggested to dismantle the ballast of a complete BioRID 50M head in order to reduce mass. Thus, the head of the BioRID P50F_V2 would be representative of a 50th percentile female by mass, while the dimensions would be somewhat too large (**Supplementary Table 1**).

When the ballast had been dismantled, the mass of the BioRID P50F_V2's head was 3.57 kg, i.e., very close to the 50th percentile female head (3.58 kg, **Supplementary Table 1**).

Supplementary Table 1. Head, lower arms and lower legs dimensions of the Hybrid III 5F, 50th percentile female, and male BioRID II.

Dimensions	Hybrid III 5F	50th percentile female	BioRID II
**Head**			
- Mass	3.73[1)] kg	3.58[3)] kg	4.54[5)] kg
- Circumference	53.8[1)] cm	54.8[3)] cm	57.15[5)] cm
- Breadth	14.2[1)] cm	14.6[3)] cm	15.5[5)] cm
- Depth	18.3[1)] cm	18.7[3)] cm	19.6[5)] cm
**Lower Arm**			
- Mass (lower arm+hand)	1.18[1)] kg	1.16[3)] kg	2.27[5)] kg
- Mass (lower arm)	0.90[1)] kg	0.85[3)] kg	1.68[5)] kg
- Mass (hand)	0.28[1)] kg	0.31[3)] kg	0.55[5)] kg
- Elbow to wrist joint	21.4[2)] cm	23.4[3)] cm	24.9[3)] cm
**Lower Leg**			
- Mass (lower leg+foot)	4.00[1)] kg	3.43[3)] kg	5.44[5)] kg
- Mass (lower leg)	3.27[1)] kg	2.82[3)] kg	3.59[5)] kg
- Mass (foot)	0.73[1)] kg	0.62[3)] kg	0.98[5)] kg
- Knee pivot height	40.6[1)] cm	45.7[3)] cm	49.3[5)] cm
- Foot breadth	8.6[1)] cm	9.2[4)] cm	9.9[5)] cm
- Foot length	22.6[1)] cm	23.5[3)] cm	25.9[5)] cm

1) Hybrid III product sheet, Humanetics Innovative Solutions, Inc.
2) Personal correspondence with Mike Beebe, Humanetics Innovative Solutions, Inc.
3) Carlsson et al. (2014)[1]
4) Young et al. (1983)[2]
5) BioRID product sheet, Humanetics Innovative Solutions, Inc.

[1] Carlsson A, Chang F, Lemmen P, Kullgren A, Schmitt K-U, Linder A, Svensson M (2014) Anthropometric Specifications, Development, and Evaluation of EvaRID—A 50th Percentile Female Rear Impact Finite Element Dummy Model. Traffic Injury Prevention, Vol 15, No. 8, pp. 855–865.

[2] Young, J.W., Chandler, R.F., Snow, C.C., Robinette, K.M., Zehner, G.F., Lofberg, M.S. (1983). Anthropometric and mass distribution characteristics of adult female body segments. Federal Aviation Administration, Civil Aeromedical Institute, Oklahoma City, OK, USA.

Simplified H-point measurement

Two new metal blocks were fixed directly to the S1 base plate (**Figure 5**), incorporating H-point tool attachments in the BioRID P50F_V2. Two vertical holes were drilled through each block, while the corresponding four threaded holes were fitted in the base plate. As can be seen in **Figure 5**, the right block is protruding slightly more relative to the base plate compared to the left block. The rear lower corner of the metal block was therefore machined to prevent it from interfering with the rubber structures in the pelvis.

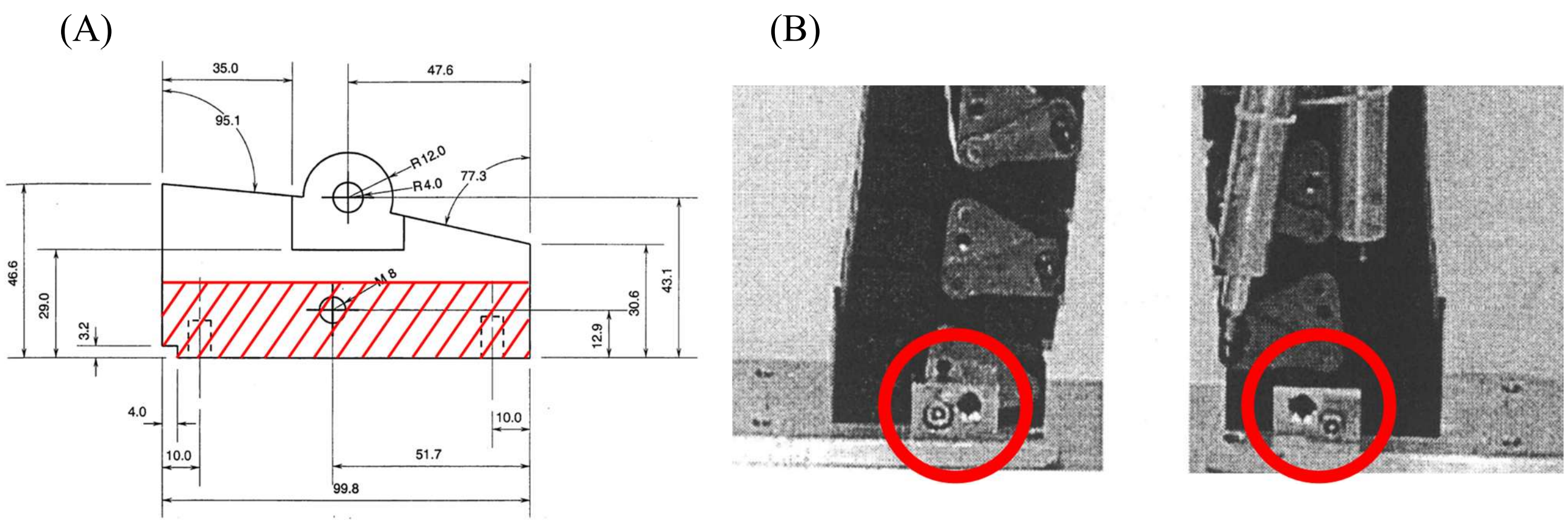

Supplementary Figure 1. A) Drawing, showing that 20 mm of the lower part of the sacral vertebra (S1) in the BioRID P50F_V1 was cut off (as indicated by parallel red lines, Carlsson et al. 2021b). Consequently, the threaded hole was removed, attaching two metal blocks (one on each side of the S1, marked with red circles). These metal blocks were penetrated by squared holes, serving as guides for the H-point measurement tool. B) The positions of the two metal blocks in the BioRID 50M dummy (left and right side, respectively).

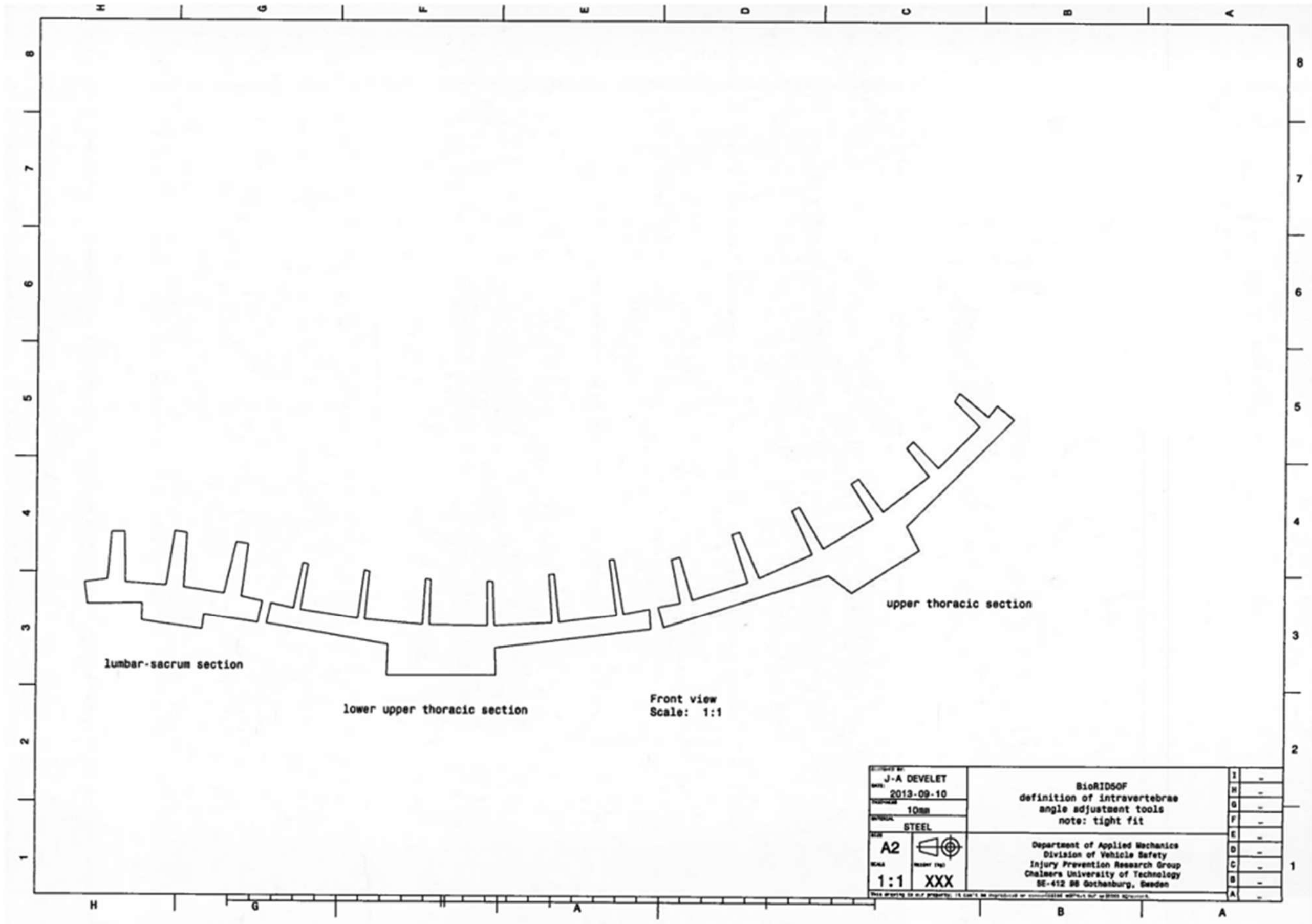

Supplementary Figure 2. Drawing of the tool used to adjust the angles between adjacent vertebrae of the BioRID P50F_V2.

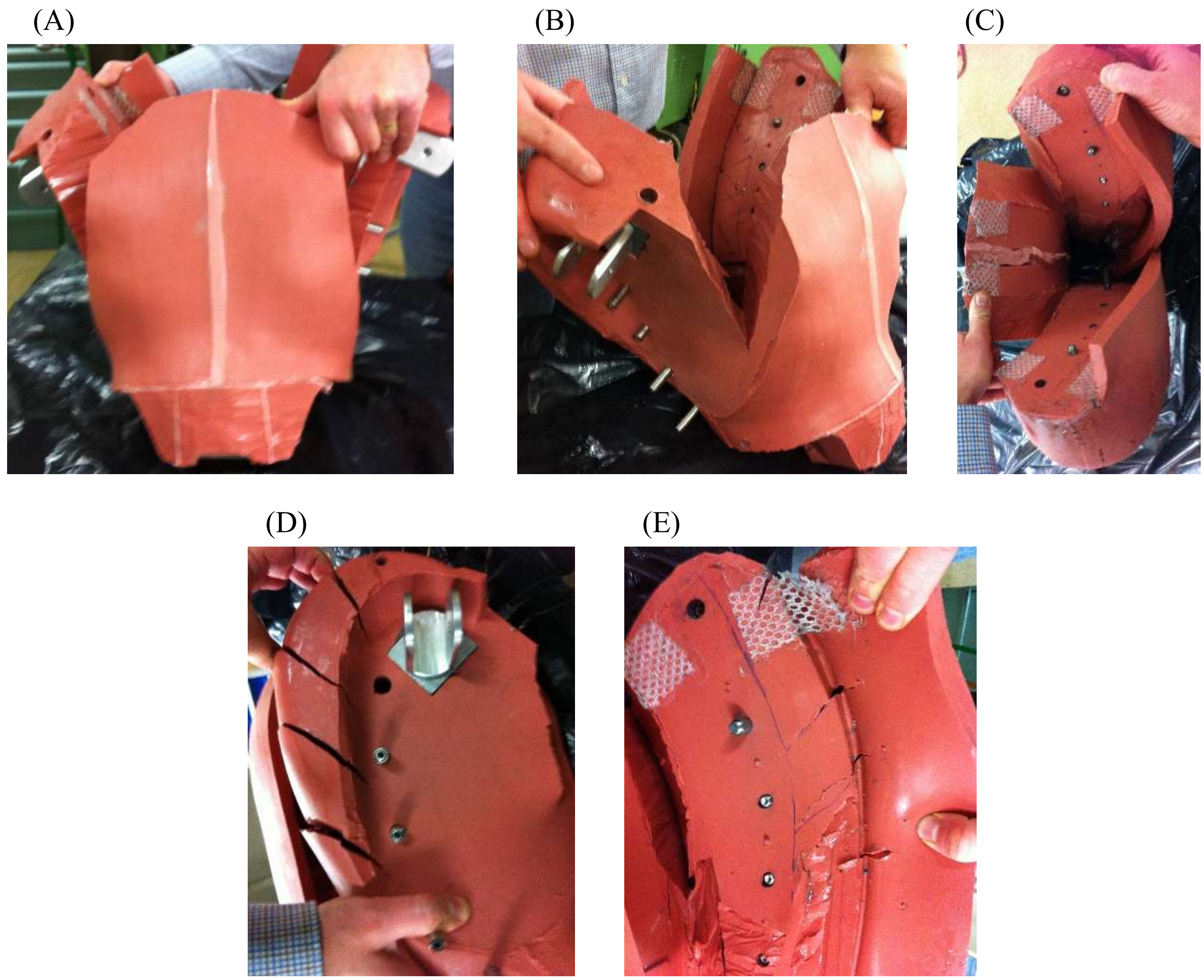

Supplementary Figure 3. Photos showing deep cuts made in the rubber torso to reduce stiffness. A) Front view. B) Side view (oblique from front). C) Top view. D) Side view (oblique from rear). E) Interior view (rear part).

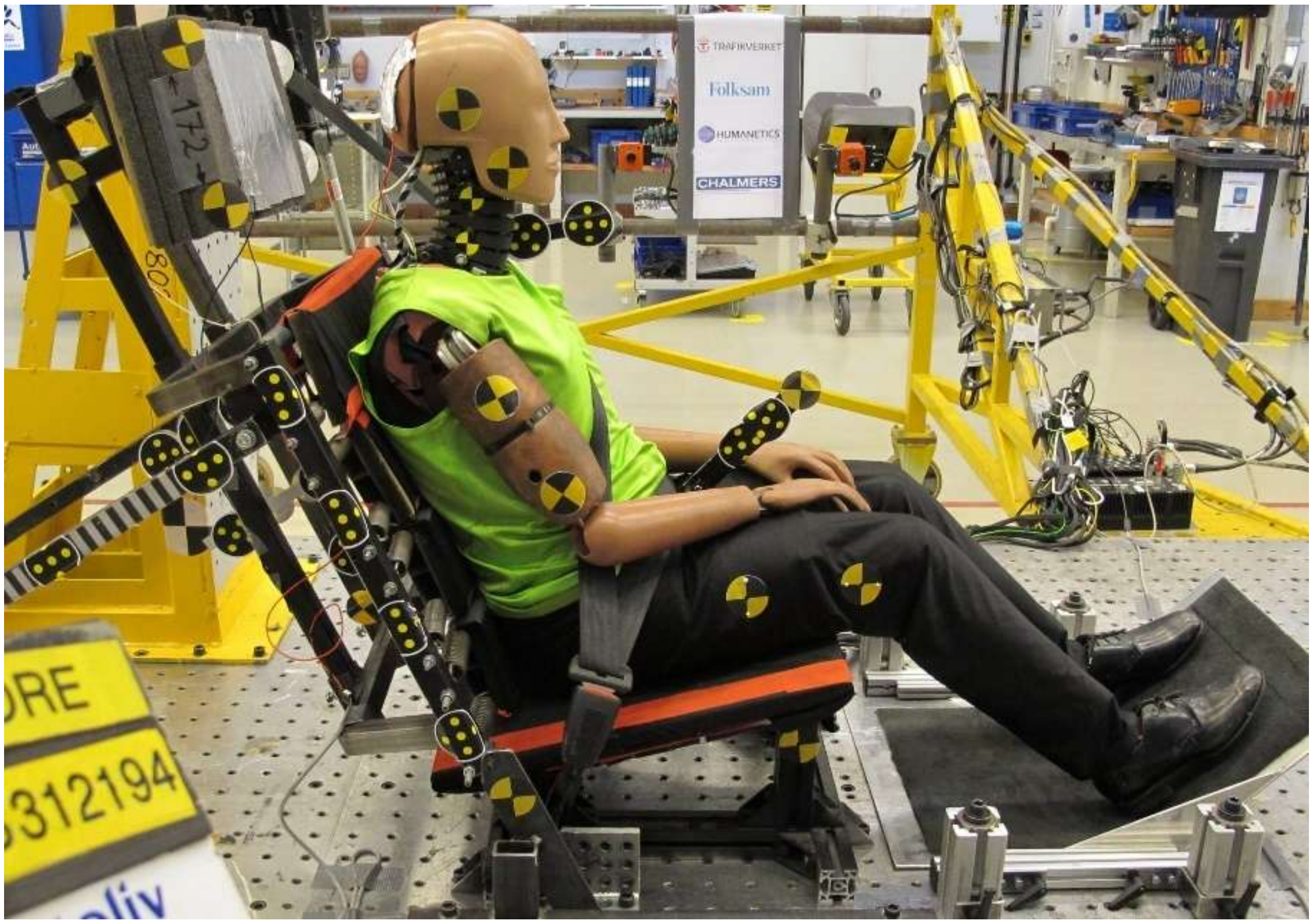

Supplementary Figure 4. Photo of the test setup for the prototype BioRID P50F_V2 in the Chalmers Laboratory Seat.
